# Application of team science best practices to the project management of a large, multi-site lung cancer screening research consortium

**DOI:** 10.1017/cts.2023.566

**Published:** 2023-06-05

**Authors:** Julie S. Steiner, Erica Blum-Barnett, Betsy Rolland, Courtney R. Kraus, Jocelyn V. Wainwright, Ruth Bedoy, Yannica Theda Martinez, Elizabeth R. Alleman, Roxy Eibergen, Lisa E. Pieper, Nikki M. Carroll, Brian Hixon, Andrew Sterrett, Katharine A. Rendle, Chelsea Saia, Anil Vachani, Debra P. Ritzwoller, Andrea Burnett-Hartman

**Affiliations:** 1 Institute for Health Research, Kaiser Permanente Colorado, Aurora, CO, USA; 2 Carbone Cancer Center and Institute for Clinical and Translational Research, School of Medicine and Public Health, University of Wisconsin, Madison, WI, USA; 3 Perelman School of Medicine, University of Pennsylvania, Philadelphia, PA, USA; 4 Center for Integrated Healthcare Research, Kaiser Permanente Hawaii, Oahu, USA; 5 Henry Ford Health System and Henry Ford Cancer Institute, Detroit, MI, USA; 6 Marshfield Clinic Research Institute, Marshfield, WI, USA

**Keywords:** Team science, cancer, consortium, project management, data management

## Abstract

Research is increasingly conducted through multi-institutional consortia, and best practices for establishing multi-site research collaborations must be employed to ensure efficient, effective, and productive translational research teams. In this manuscript, we describe how the Population-based Research to Optimize the Screening Process Lung Research Center (PROSPR-Lung) utilized evidence-based Science of Team Science (SciTS) best practices to establish the consortium’s infrastructure and processes to promote translational research in lung cancer screening. We provide specific, actionable examples of how we: (1) developed and reinforced a shared mission, vision, and goals; (2) maintained a transparent and representative leadership structure; (3) employed strong research support systems; (4) provided efficient and effective data management; (5) promoted interdisciplinary conversations; and (6) built a culture of trust. We offer guidance for managing a multi-site research center and data repository that may be applied to a variety of settings. Finally, we detail specific project management tools and processes used to drive collaboration, efficiency, and scientific productivity.

## Introduction

Multi-site research collaborations have grown substantially over the past three decades and are now the norm in many areas of clinical and translational research [1]. As these collaborations increase, so does the need for scientific research teams to implement processes and procedures that foster collaboration, efficiency, and productivity. Recognizing unique challenges faced by translational research teams (e.g., operational inefficiency, data incompatibility, and federated human subjects oversight) [2], the growing field of the Science of Team Science (SciTS) has developed the following evidence-based best practices [3]: (1) ensure the team has a shared mission, vision, and goals; (2) develop a transparent and representative leadership structure; (3) employ strong research support systems; (4) provide efficient and effective data management; (5) promote interdisciplinary conversations on approaches, methods, and results; and (6) build a culture of trust. Current literature lacks specific examples of the application of these best practices to the management of multi-site translational research programs.

Using SciTS best practices as our framework, we describe the structure and processes employed to manage a large, multi-site translational research center and data repository: the Population-based Research to Optimize the Screening Process (PROSPR) Lung Research Center (PROSPR-Lung) [4]. Launched in 2018 as part of the National Cancer Institute’s (Bethesda, MD) PROSPR II Consortium [5], PROSPR-Lung includes five heterogeneous healthcare systems: Henry Ford Health (HFH), Kaiser Permanente Colorado (KPCO), Kaiser Permanente Hawaii (KPHI), Marshfield Clinic Health System (MCHS), and the University of Pennsylvania Health System (UPHS). PROSPR-Lung’s aims are to (1) build a large, multi-site data repository relevant to lung cancer screening (LCS) research, (2) conduct observational studies on LCS uptake, harms, costs, risks, and the use of tobacco cessation strategies in the context of screening, (3) identify opportunities for interventions to optimize LCS, and (4) participate in research involving other cancer screening centers within the PROSPR II consortium [5] (i.e., Trans-PROSPR research).

When establishing large research consortia, prioritizing and resourcing project coordination and management from the outset clears the way for researchers to focus on science [6]. To this end, the PROSPR-Lung infrastructure applies team science best practices to promote effective collaboration and streamline processes for administrative and data management. In this manuscript, we demonstrate how PROSPR-Lung implements these principles, and we provide guidance for managing a multi-site research center and data repository that is applicable to a variety of academic and community-based health system research settings.

## SciTS best practices translated into specific research project management strategies

**Figure 1.**
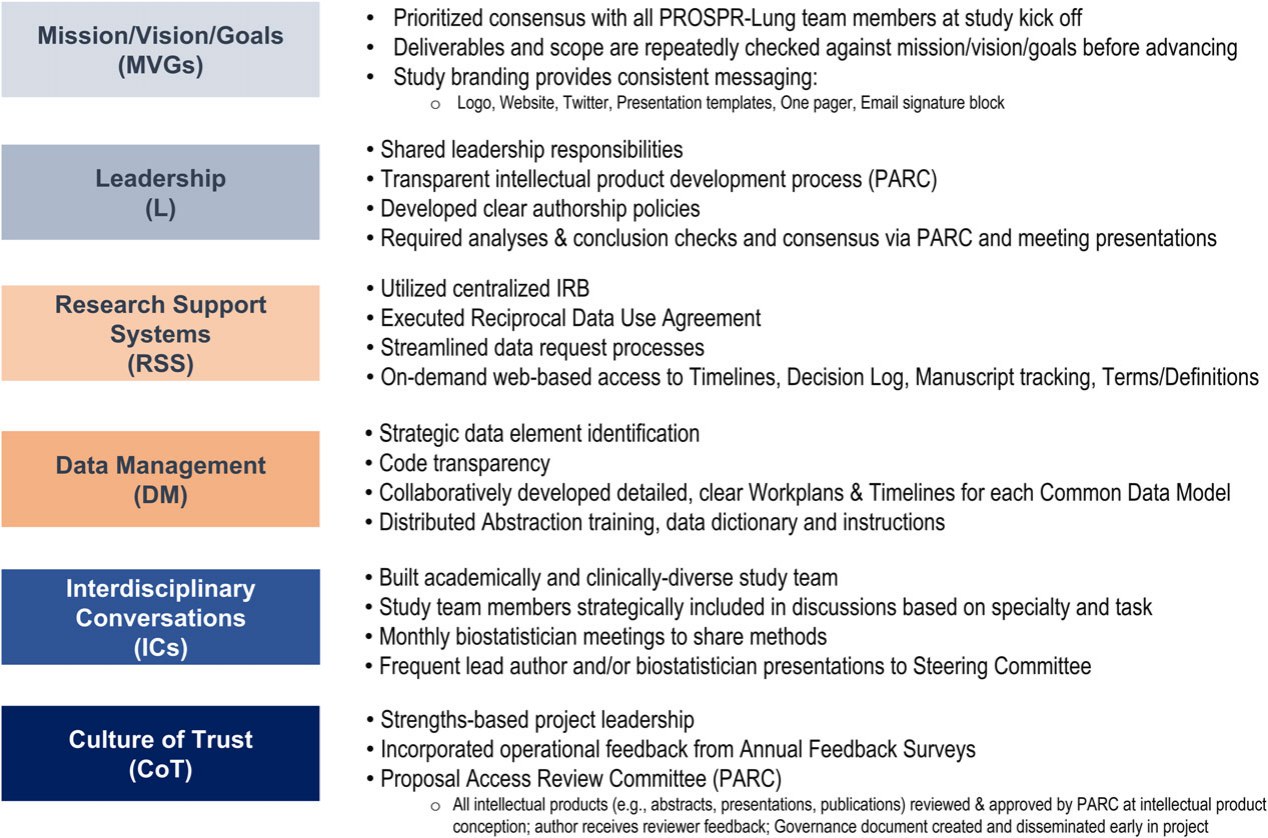
PROSPR-Lung strategies to implement SciTS best practices. IRB = Institutional Review Board, PARC = Proposal Access Review Committee, SciTS = Science of Team Science.

Rolland *et al*. [3] maps the intersection of six SciTS best practices for scientific reproducibility with Begley *et al*.’s [7] six Good Institutional Practices, providing the opportunity to assess a team’s performance based on specific behaviors. In this section, we apply six SciTS best practices to PROSPR-Lung’s infrastructure and highlight specific strategies used to stimulate and support a high-performing team environment (Fig. 1).

### Shared mission, vision, and goals

Shared goals are a critical component of interdisciplinary teamwork, and gaining team consensus on shared goals is one of the first objectives that should be accomplished [8,9]. While high-level research goals are often predetermined in the funding announcement, developing a mission, vision, and specific goals with the study team from the start creates a solid foundation to assess deliverables and scope over the course of a project. To ensure team members had awareness of and agreement with the PROSPR-Lung mission, vision, and goals, a virtual “Project Launch” meeting was held for all study team members at each site (e.g., Investigators, Project Managers, Programmers, Biostatisticians, Research Assistants, and more). The launch meeting provided a space for team members to discuss, critique, and refine the mission, vision, and goals, which were further solidified at PROSPR-Lung’s initial in-person meeting. Subsequently, PROSPR-Lung leadership took steps to guarantee the mission, vision, and goals remain a focal point in meetings, communications, and project deliverables. For example, when an individual requests the use of PROSPR-Lung data for an abstract, manuscript, or new funding application proposal, the requestor must specify how the research contributes to PROSPR-Lung’s mission.

Early in year 2, PROSPR-Lung created a logo, website [10], and Twitter account [11] to further unite around a team identity and to provide cohesion for the team [12]. To promote consistency in dissemination efforts, PROSPR-Lung created branded posters and presentation templates and made them available, for optional use, to all team members on the consortium’s web-based document-sharing platform.

Keeping PROSPR-Lung’s shared mission, vision, and goals at the center of our work provides the study team with guidance and insight into how their work contributes to study results and to the scientific community. It has allowed project leadership to maintain a delicate but critical balance between achieving individual and research team goals and ensuring that the main results advance together.

### Leadership

Strong leadership is noted throughout SciTS literature [3,13–15] as a necessity for building and maintaining a successful, productive research team. Study leadership is determined during the application development phase when roles and structures are strategically designed to set the study up for success. PROSPR-Lung designed a leadership structure (Appendix 1) that includes study team members from across sites and roles consisting of a small governing body (Executive Committee) responsible for overall PROSPR-Lung guidance and oversight, a larger governing body (Steering Committee) responsible for making strategic decisions about the overall scientific direction of PROSPR-Lung, and three operational cores (Administrative Core, Data Acquisition Unit [DAU], and Dissemination and Engagement Core). The research center’s governing bodies are composed of representatives from each site, providing diversity in knowledge, specialization, and experience. Specific research project leadership within PROSPR-Lung is divided among Site Investigators in alignment with their expertise. Throughout PROSPR-Lung, successful collaboration is promoted through strengths-based division of labor, with responsibilities and accountability assumed not simply by role but also by expertise, interest, passion, and career goals.

**Figure 2.**
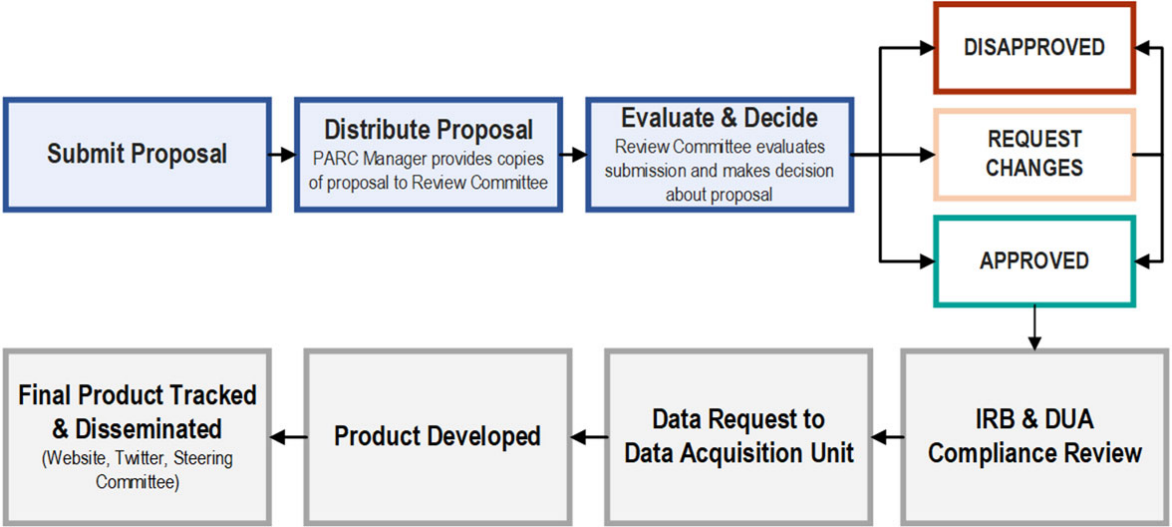
PROSPR-Lung’s Proposal Access Review Committee (PARC) intellectual product proposal review, administrative core compliance review, data request, and tracking process. DUA = Data Use Agreement, IRB = Institutional Review Board, PARC = Proposal Access Review Committee.

In addition to shared leadership and accountability, Rolland *et al*. describe high-functioning teams as those with written authorship and operational policies [3]. Explicit, written governance, and authorship guidelines were among the early priorities of the PROSPR-Lung Administrative Core and are adhered to at all levels of leadership within the study team. The leadership structure also fostered responsible, compliant, transparent data use and analyses through our Proposal Access Review Committee (PARC) (Fig. 2). The PARC, detailed below, solicits lead author presentations to the Steering Committee and acts as the hub for review, tracking, and dissemination of PROSPR-Lung intellectual products.

PROSPR-Lung’s leadership strategy placed inclusion, strengths-based leadership, and transparency at its core. In addition to fostering productivity, these PROSPR-Lung leadership elements help to establish and maintain a culture of trust that is essential for effective teams.

### Research support systems

**Figure 3.**
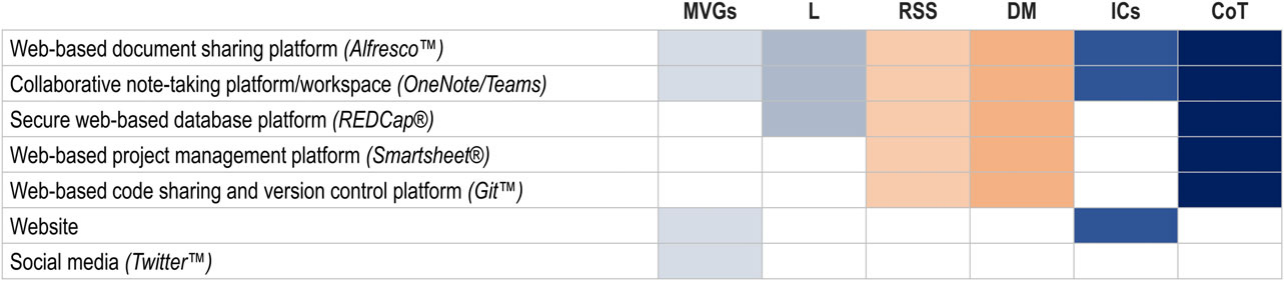
Project management tools utilized by PROSPR-Lung to drive collaboration, efficiency, and scientific progress. Refer to Fig. 1 for specific SciTS best practices for which these tools were used to implement. CoT = culture of trust, DM = data management, ICs = interdisciplinary conversations, L = leadership, MVGs = mission, vision, and goals, RSS = research support systems.

A research support system is the infrastructure in which the project and its staff, data, communications, and science can be coordinated, managed, and disseminated. Rolland *et al*. highlight research support systems as the singular best practice that “strongly influence[s]” all other best practices [3]. As with the study leadership plan, the formulation of PROSPR-Lung’s research support system began at the proposal development phase and was reviewed and reworked to ensure efficiency and necessity throughout the award period. PROSPR-Lung’s key research support system elements and implementation prioritization strategy is detailed below, summarized in Fig. 3, and presented chronologically in Appendix 2.

#### Human subjects compliance

In accordance with the National Institute of Health’s policy NOT-OD-16-094 [16], PROSPR-Lung utilizes a centralized Institutional Review Board (IRB) to manage human subjects compliance. The advantages of a centralized IRB to which all sites cede authority for review and oversight include efficiency, standardized and consistent compliance review, and transparency. Working closely with site Project Managers, the Administrative Core used templates to obtain site information and ceding documentation, which resulted in the efficient establishment of centralized IRB management for PROSPR-Lung.

#### Data Use Agreement

The development and execution of a Reciprocal Data Use Agreement was the first major lift of the Administrative Core and involved months of conversations with Research Compliance, Sponsored Projects Administration, Site Principal Investigators, Biostatisticians, and Project Managers. The framework of the PROSPR-Lung Reciprocal Data Use Agreement was modeled after previously developed consortium agreements [17,18], including agreements used in the HealthCare Systems Research Network (HCSRN) [19,20]. The PROSPR-Lung Reciprocal Data Use Agreement details the data elements that all sites agree to share between each other and specifies what can or cannot be shared with the funder and the funder’s contracted data management group. Clearly detailing and agreeing on what data can be exchanged with whom, in one referential document, was the essential first step in creating a process that ultimately allows PROSPR-Lung datasets to be exchanged in a compliant and efficient manner.

#### Communication infrastructure

The PROSPR-Lung Administrative and DAU Cores use various tools to streamline communications and track progress (Fig. 3). In order for tools to be successful in overcoming communication barriers, they need to be centrally managed, readily available and permissible for all sites to use, present no cost burden to sites, easy to use for on-boarding and off-boarding team members, and have an effective user interface that eliminates the need for onerous training.

At the lead site, the Administrative and DAU Cores facilitate collaboration via their intranet system and institutionally supported software (SharePoint® and Teams®) [21,22] and use a shared digital notebook (OneNote®) [23], to streamline topic tracking, agenda creation, note-taking, and action item management across multiple meetings and topics. Since most PROSPR-Lung sites are HCSRN members, we chose a web-based document management platform available via HCSRN (Alfresco™) [24], at no cost to the sites, to store important up-to-date documents and reduce unnecessary email correspondence. The password-protected PROSPR-Lung content management site houses documents that need to be available to all sites on demand, including the Reciprocal Data Use Agreement, human subjects review approvals, PARC governance documents, data dictionaries, and other instructional or procedural documents. It also provides instant access to the study meeting minutes, contact list, presentation template, hyperlink to the PARC proposal form, current list of all PROSPR-Lung scientific products, and relevant lung cancer literature. Storing these items on a secure, web-based document management site improves efficiency by empowering sites to access study documents independently.

The primary project management tool used within PROSPR-Lung is a web-based platform that allows licensed individuals to create workspaces and processes, and provides users with access to read-only or data entry sheets (SmartSheet®) [25]. PROSPR-Lung utilizes this platform to provide all study team members with on-demand access to key information such as data acquisition timelines and schedules, workplan progress, decision log entries, and manuscript statuses. The Administrative Core also uses the platform for finance management, human subjects review tracking, task management, and programmed reminders to revisit topics at a more propitious time.

PROSPR-Lung’s leadership prioritized implementation of strong research infrastructure by directing significant resources to its development. In the first 2 years of the project, approximately 1.75 full-time equivalent (FTE) effort was directed to two Project Managers (one Lead and one DAU), two Research Specialists, and one Research Assistant at the lead site to develop and implement PROSPR-Lung’s infrastructure, as well as approximately 0.50 FTE to UPHS’ Project Manager as PARC was collaboratively developed and then managed by UPHS. Strategically directing resources to the development of a strong research infrastructure promoted efficient IRB management, compliant data exchanges, effective communication, and ongoing transparency for all study staff. These practices, in turn, contribute to meeting study deliverables and maintaining a culture of trust.

### Data management

The efficient data management system implemented and improved throughout the study complements the key elements of PROSPR-Lung’s research support system. A strong research data management system requires a distinct project management and leadership structure, a clear and thorough Data Use Agreement, programming code transparency, detailed workplans, documentation of variable definitions and other important details within a data dictionary, consistent file nomenclature, and excellent communication.

The PROSPR-Lung DAU is responsible for the creation and maintenance of an accessible and transparent data management system for all team members. It aims to identify data elements needed for PROSPR-Lung analyses, develop and refine a data repository/common data model (CDM) with data from all sites, and provide a limited subset of PROSPR-Lung data for external use [26]. In line with best practices, the PROSPR-Lung team identifies the questions the data are expected to answer and the data needed to answer the questions, assesses availability of the data, develops common data elements, maps and transforms individual data points to common data elements, and performs quality checks (Appendix 3) [27]. Further, PROSPR-Lung’s data management system includes an analytic dataset request process with built-in compliance checks (Fig. 2). In the first 2 years of the project, approximately 4.0 FTE was directed to the DAU’s Project Manager, Data Specialists/Programmers, and Biostatisticians at the lead site as infrastructure was developed and implemented.

#### Data extraction, programmer codes, and workplans

**Figure 4.**
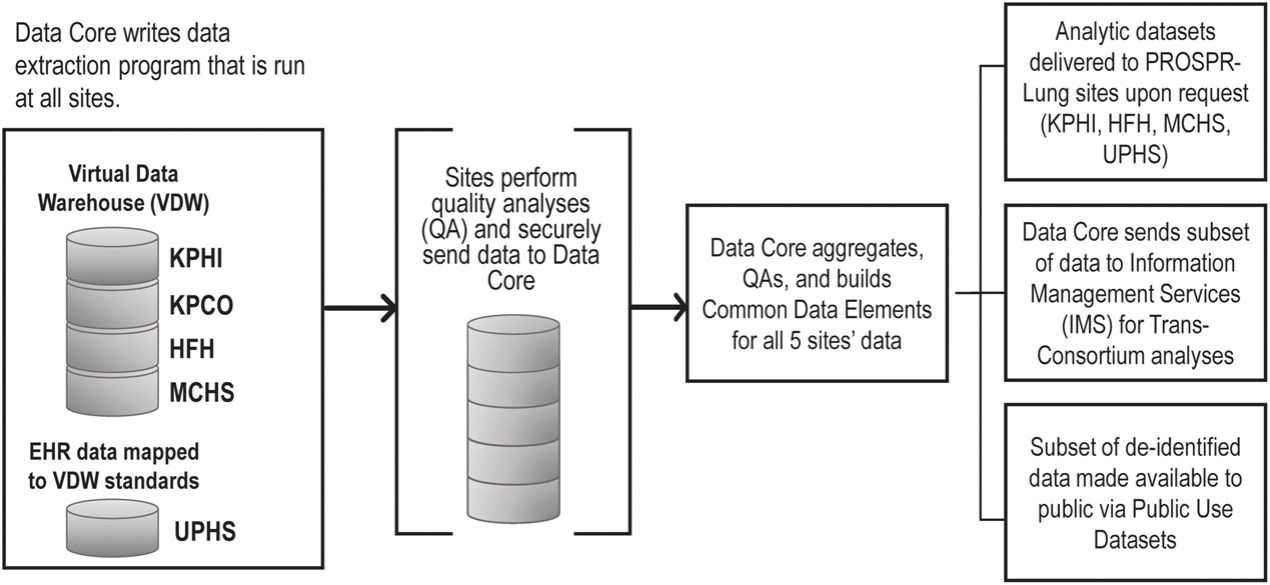
PROSPR-Lung’s methodology to capture, harmonize, and provide limited and deidentified datasets. EHR = electronic health record, HFH = Henry Ford Health, KPHI = Kaiser Permanente Hawaii, KPCO = Kaiser Permanente Colorado, MCHS = Marshfield Clinical Health System, NCI = National Cancer Institute, UPHS = University of Pennsylvania Health System.

Keeping its mission, vision, and goals at the center of decision-making, PROSPR-Lung executes the iterative process of determining which variables need to be captured and made available to fulfill the project’s goals. At the same time, the DAU leads collaboration with study sites to create and validate the data collection and database creation process (Fig. 4). Focusing on flexibility, the DAU built on the existing infrastructure of HCSRN’s Virtual Data Warehouse (VDW) [19,28] to create a relational database with standardized data elements. This allows PROSPR-Lung to meet project aims while also setting up its database to readily answer future research questions.

To extract the data elements, the DAU develops programming code centrally, tests the code at one subsite, and develops workplans with programming code. These workplans are distributed to all subsites and recorded in SmartSheet®. On bimonthly conference calls, the DAU facilitates communication across site programmers to address challenges associated with current and future workplans, update site progress, identify issues, and work toward resolution. Code version control is maintained using a distributed version control system for tracking changes in source code (Git™) [29] and pushed to a remote repository (GitLab™) [30] to improve transparency and collaboration with sites during code distribution. A rigorous three-round quality assurance process is administered by the DAU before finalizing each CDM (Appendix 3).

#### Data abstraction

Manual data abstraction is required for a subset of variables to fill in missing data or provide quality assurance. The Administrative Core, DAU, and Investigators developed the list of variables required to meet specific analytic needs. Importantly, the Investigator team includes clinicians who provide standard definitions and interpretation of clinical data acquired through abstraction. Based on learnings from prior studies, abstraction templates are developed centrally, programmed in a secure, web-based software platform designed to support data capture for research studies (Research Electronic Data Capture [REDCap]®) [31], pilot-tested, and then distributed to each site along with a detailed manual containing abstraction instructions, processes, and definitions of LCS data. Coupled with recorded training sessions, these resources facilitate high inter-rater reliability and are also used to train new study team members.

#### Fulfilling analytic dataset requests

PROSPR-Lung devised a data request process where individuals request analytic datasets from the DAU via a Data Request Form in REDCap® (Appendix 4). Data Request Forms are linked to the corresponding PARC proposal in REDCap®, thus streamlining internal review. When a Data Request Form is submitted, a programmed alert is delivered to the DAU, which triggers triage and ad hoc meetings between the DAU and requestor to discuss parameters and address questions or challenges. Hidden administrative fields allow the DAU to create tracking reports and ensure fulfillment.

Successfully harmonizing a large amount of complex data across multiple sites with unique data systems presents many challenges, among them working together toward a common goal, with each site moving at a different pace. Building a data management system with clear workplans, on-demand visibility into current and future workplans via a web-based project management tool, consistent data quality checks, frequent meetings to discuss issues, a transparent data request process, and a robust data sharing policy allows PROSPR-Lung to effectively pursue project deliverables.

### Interdisciplinary conversations on approaches, methods, and results

As defined by Choi and Pak, “interdisciplinarity analyzes, synthesizes, and harmonizes links between disciplines into a coordinated and coherent whole [9].” PROSPR-Lung cultivated an interdisciplinary team that includes expertise in pulmonology, radiology, health economics, epidemiology, biostatistics, statistical modeling, bioinformatics, health equity research, behavioral health, and dissemination and implementation research. Study team members are strategically included in meetings based on specialty, task, and interest. Biostatisticians meet monthly to share methods and obtain feedback on analytic approaches. Leaders to move forward publications, presentations, and other intellectual products are determined based on strengths and interests. Monthly Steering Committee meetings provide opportunities to review current manuscript proposals, analysis plans, and results, with the benefit of multidisciplinary perspectives united in a common goal. Semiannual in-person or virtual meetings provide focused time for study team discussions on science, methods, results, and learnings. Steering Committee and semiannual meetings also provide the opportunity to collaborate with scientists from NCI, other Trans-PROSPR members, and subject matter experts invited as guest speakers.

Prioritizing interdisciplinary discussions facilitates study team engagement and valuation, and results in better research products by establishing accurate definitions and employing data quality processes early in development [3].

### Culture of trust

Building and maintaining trust that spans time and distance requires psychological safety, which includes transparency, visibility, accountability, and inclusivity [13]. Teams that trust each other feel comfortable questioning each other’s data, share mistakes and misunderstandings, encourage open discussions, meet commitments, and trust that other team members will do the same. A team that has cultivated a culture of psychological safety also encourages critical review of research proposals and analytic conclusions [3]. To facilitate these imperative team assets, PROSPR-Lung prioritized the development of the PARC early in the project timeline (Appendix 2). The PARC establishes and oversees fair, clear, and efficient policies for all PROSPR-Lung intellectual products. These products are defined as publications, meeting abstracts, presentations, or new funding proposals that involve PROSPR-Lung investigators or staff who use funds and/or data from one or more PROSPR-Lung sites. PROSPR-Lung’s detailed governance document, modeled after the PROSPR I governance document, specifies policies and procedures for the review and approval process. This governance document also serves as a central point of reference for authorship requirements and responsibilities, adapted from the International Committee of Medical Journal Editors’ recommendations [32]. PROSPR-Lung’s guidelines require that at least one person from each data-contributing site be included as a coauthor to ensure accurate interpretation and provide key insight into the idiosyncrasies of their site’s data. Rotating PROSPR-Lung site members are responsible for reviewing all proposals for significance, design, feasibility, and potential overlap with other projects, as well as for making recommendations about all proposals. A Project Manager oversees the PARC process (Fig. 2), manages communications between proposal authors and PARC members, and facilitates meetings to discuss procedural issues and resolve disputes. REDCap® [31] is used to manage intellectual product submission (Appendix 5), review, approval (Appendix 6), and tracking forms.

Cultivating a culture of trust is a central thread woven throughout the SciTS best practices that PROSPR-Lung applies to its operations and demonstrates in essential ways, including: (1) thoughtfully planning and strategically executing meetings that include salient people and topics; (2) distributing detailed meeting minutes in a timely manner with action items, deadlines, and assigned personnel to ensure accountability; (3) establishing data delivery timelines and workplans with web-based centralized progress tracking in close collaboration between the DAU and the data-contributing sites; (4) uploading final products to (i) the public PROSPR-Lung website [10], (ii) a secure, web-based document management platform [24] provided by HCSRN [33], and (iii) our internal product tracking system in REDCap® [31]; and (5) formally soliciting site feedback through annual surveys (Appendix 7 and 8), presenting results to the Steering Committee, and incorporating feedback to improve study operations. Propelled by suggestions in annual survey responses, PROSPR-Lung implemented role-specific meetings to ensure all voices were heard, improved workplan clarity and consistency of code repository use, incorporated site programmer feedback of draft workplans prior to release, and improved action item communications by including action item tables in emails as well as in meeting minutes. These strategies to establish a culture of trust helped PROSPR-Lung build a consortium that values transparency and encourages sharing new ideas.

## Discussion

Our paper highlights the importance of intentionally and methodically prioritizing project management that applies SciTS best practices to establish translational research consortia, and throughout study implementation, to facilitate high-impact research. We also discuss some of the barriers, such as variations in permissions that affect use of project management tools, and facilitators, such as strong project leadership structures, to establishing research infrastructure.

PROSPR-Lung developed and maintains a strong leadership structure that promotes open communication, trust, and transparency. We demonstrate that there are many opportunities throughout a study to promote shared mission, vision, and goals, including study branding, consistent messaging, and inclusive meetings. Strategically building interdisciplinary teams and including study staff in discussions based on specialty and task promote engagement and efficiency. Encouraging investigators and research staff to lead facets of the consortia based on their personal areas of expertise and interests increases efficiency and helps build a culture of trust.

In addition to strengths-based project assignments, our consortium took specific actions to remove barriers in multi-site translational research, including utilizing a centralized IRB to which sites ceded, executing a Reciprocal Data Use Agreement, and building a centralized process for reviewing and tracking intellectual products. Managing intellectual projects through the PARC process from start to finish and providing regular updates to the Steering Committee, allows the PROSPR-Lung consortium to stay abreast of potential proposal conflicts or overlaps, manage challenges with analytic plans, ensure compliance with the IRB, and demonstrate productivity. Establishing infrastructure and policies at the beginning of the study allows us to provide clear guidance in the event of procedural issues. Having a centrally located DAU and Administrative Core facilitate effective governance and infrastructure management, prioritization of data element capture, creation and distribution of workplans, and efficient management of timelines and deliverables. By applying key SciTS principles to our project coordination and management, PROSPR-Lung’s investigators can direct more time to conduct rigorous, translational science, which also provides opportunities for early-career investigators and physician-scientists to lead research [34,35]. PROSPR-Lung accomplishes these goals by building trust and confidence among team members, using centrally managed, evidence-based governance policies, and following team science best practices at each stage of our study.

## Future directions

Although some aspects of this theoretical framework pertain specifically to human subjects research, the principles discussed herein could apply to a wide variety of teams in professional settings (e.g., critical care and business innovations [36,37]). Future research should continue to explore knowledge transfer between settings in an ongoing effort to promote development of strong project teams and infrastructure.

As Allmaras *et al*. [38] observe, it is difficult but important to evaluate the success of large research consortia, such as PROSPR-Lung, especially as funding for collaborative award mechanisms increases. Having built and implemented the PROSPR-Lung infrastructure, we will continue to assess the impact of this theoretical construct on our scientific and organizational progress, as well as scientific and clinical investigator burden reduction achieved through devoting noninvestigator personnel time to its implementation. We will assess impact by soliciting feedback from consortium members through surveys and structured conversations, as well as by collecting data on outputs (e.g., publications and ancillary grants) and outcomes (e.g., policy changes). In doing so, we will continue to improve the management of the PROSPR-Lung consortium and its future endeavors and further contribute to the SciTS and research project management knowledge base.

## Supplementary material

For supplementary material accompanying this paper visit https://doi.org/10.1017/cts.2023.566. click here to view supplementary material
