# Supplementary material for: Application of team science best practices to the project management of a large, multi-site lung cancer screening research consortium
Source: J Clin Transl Sci. 2023 Jun 5;7(1):e145. doi: 10.1017/cts.2023.566 (PMC10346083; doi:10.1017/cts.2023.566)
Supplement: Supplementary file 1 [file S2059866123005666sup001.docx]

Appendix 1: PROSPR-Lung Governance Model


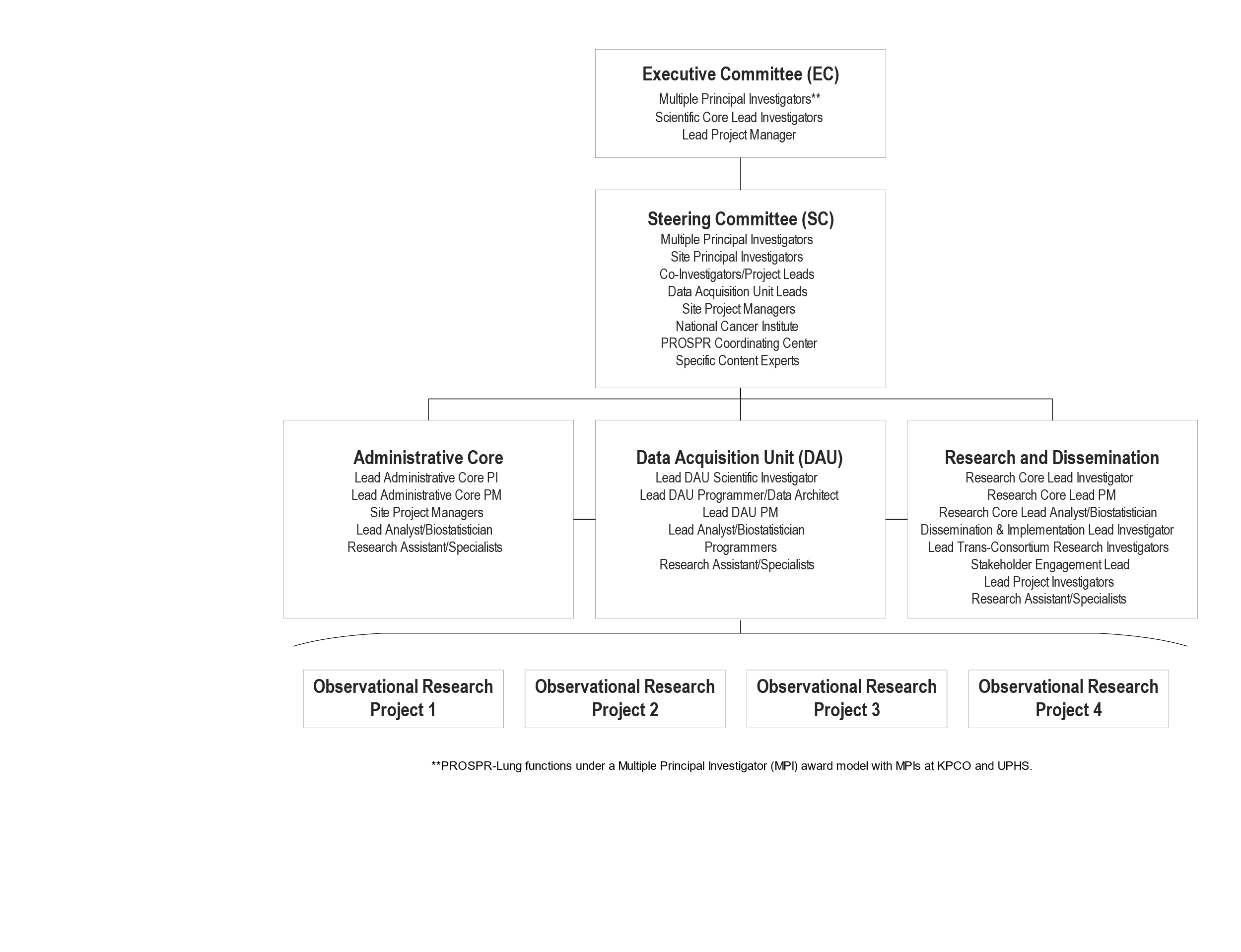


Figure Legend: This figure illustrates PROSPR-Lung’s shared governance, distributed leadership, and inclusion strategy that enabled transparency, visibility, and interdisciplinary conversations. PI = Principal Investigator. PM = Project Manager.

Appendix 2: PROSPR-Lung infrastructure timeline


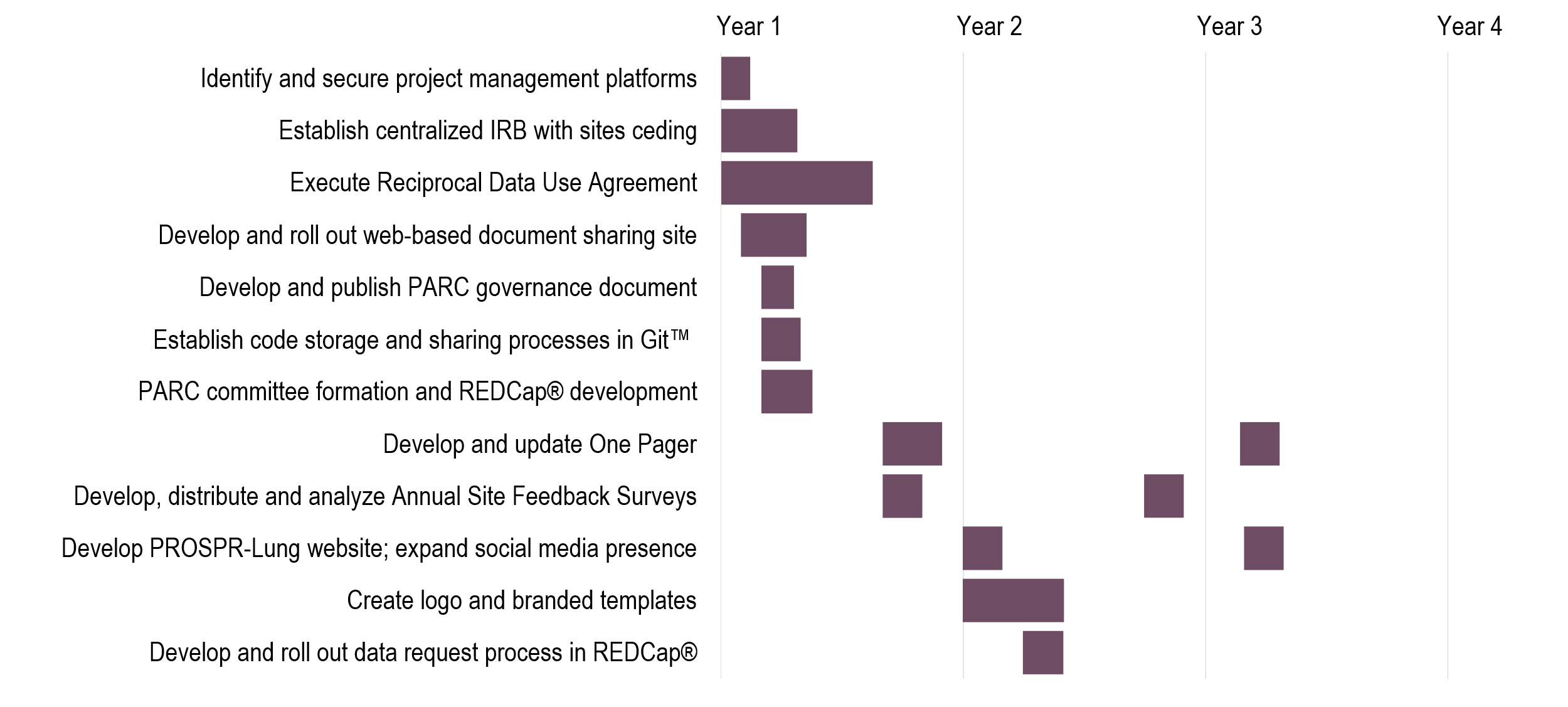


Figure Legend: This figure reflects the order that PROSPR-Lung implemented key elements of the research infrastructure. IRB = Institutional Review Board. PARC = Proposal Access Review Committee. REDCap^®^ = Research Electronic Data Capture^®.^

Appendix 3: PROSPR-Lung Common Data Model development, execution, and quality assurance process


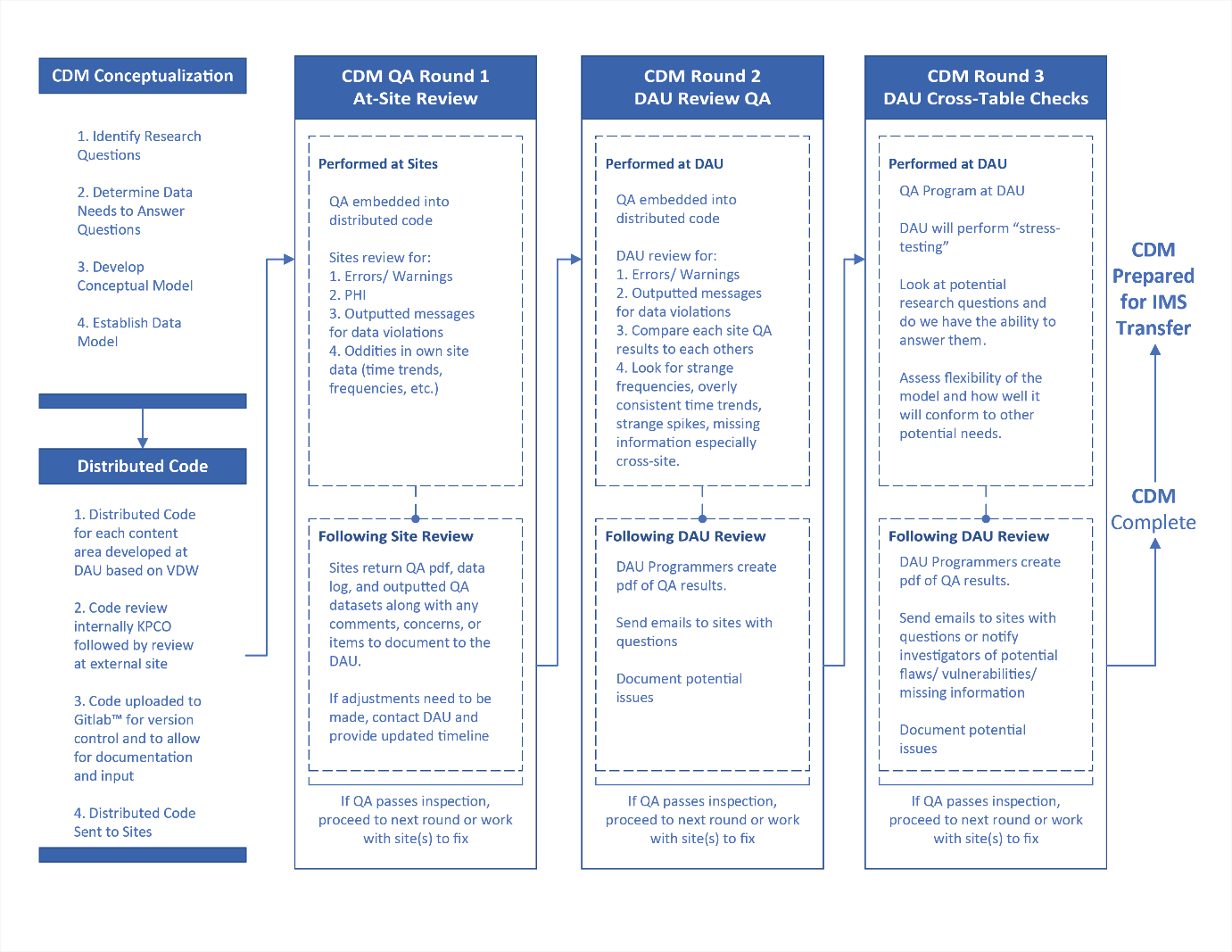


Figure Legend: This figure details the development of PROSPR-Lung’s Common Data Model (CDM) from conception to data transfer. The Data Acquisition Unit (DAU) administers a rigorous, consistent, three-round quality assurance process. Quality assurance (QA) checks are embedded in the distributed code, such that each site can review their data for errors and potential Protected Health Information (PHI) violations before transmitting datasets to the central data repository at the DAU. Upon receipt of the data, the DAU performs similar site-specific quality assurance checks, followed by cross-table checks across all PROSPR-Lung sites. This thoroughly documented multi-site quality assurance process provides the DAU and site programmers with multiple opportunities to ensure datasets are compliant, accurate, and comprehensive.

Appendix 4: PROSPR-Lung Data Request Form (DRF)

The Data Request Form (DRF) was programmed into REDCap^®^ and linked to an individual’s Proposal. After receiving Proposal Access Review Committee (PARC) approval to use PROSPR-Lung data (Figure 2 in the manuscript), the Project Manager releases this form to the requestor. Because the form is linked to the Proposal in REDCap^®^, much of the basic project information is programmed to populate into the form, thus saving time for the requestor. Data is formally requested through the completion and submission of this form in REDCap^®^.

**Data Request Form**

This form was designed to request data for the analytic plan that you created for [insert project title].

As you fill out this form, we recommend you save your progress by clicking on the "Save & Return Later" button. This will save your responses and allow you to leave the form and return to it at a later time. When you are ready to submit the completed form, click on the "submit" button. After you press the "submit" button, you cannot go back and change your responses.

Please reach out to [DAU lead] with any questions you may have while filling out this form. Once this form has been submitted, it will be reviewed by the DAU and a DAU team member will contact the Project Lead and Lead Statistician/Analyst within 5 business days.

| **BASIC PROJECT INFORMATION** |
| --- |

| Today’s Date | _________________________________ | |
| --- | --- | --- |
| Please indicate who is completing the form:  This form should be completed by the project lead and/or the project statistician/analyst. Check all that apply. |  | Project Lead |
|  |  | Project Statistician/Analyst |
| Please enter the name of the Project Statistician/Analyst: | _________________________________ | |
| Please enter the email address for the Project Statistician/Analyst: | _________________________________ | |
| Project Title: [Project title piped in from proposal] | _________________________________ | |
| Project Lead First Name: [Project lead first name piped in from proposal] | _________________________________ | |
| Project Lead Last Name: [Project lead last name piped in from proposal] | _________________________________ | |
| Project Lead Email: [Project lead email address piped in from proposal] | _________________________________ | |
| Project Lead Affiliation: [Project lead affiliation piped in from proposal] | _________________________________ | |
| Specific Aims: [Specific aims and objectives piped in from proposal] | _________________________________ | |
| Background/Rationale/Significance: [Background/Rationale/Significance piped in from proposal] | _________________________________ | |
| Study Type: [Study Type piped in from proposal] | _________________________________ | |
| Methods: [Methods outline piped in from proposal] | _________________________________ | |
| Name of person to receive data:  (data will be transferred via Secure File Transfer) | _________________________________ | |
| Email address of person receiving data:  (data will be transferred via Secure File Transfer) | _________________________________ | |
| Date Data Needed:  Please allow up to 6 weeks to receive data. Timing of the delivery of data sets may vary based on the specifics of your request and the current workload of the DAU Core. | _________________________________ | |

| **ANALYTIC PLAN** |
| --- |

| Populations/Members to Include:  Check all that apply |  | Site 1 |
| --- | --- | --- |
|  |  | Site 2 |
|  |  | Site 3 |
|  |  | Site 4 |
|  |  | Site 5 |
|  |  | All Populations/Members |
| Study Inclusion Criteria  Please be as specific as possible. | _________________________________ | |
| Study Exclusion Criteria  Please be as specific as possible. | _________________________________ | |
| Time Period of Data to Include:  Please format as: mm/dd/yyyy - mm/dd/yyyy | _________________________________ | |
| Outcomes: | _________________________________ | |
| Exposures, covariates, confounders, adjustment variables, independent variables of interest: | _________________________________ | |
| Statistical Methods for all Aims:  Copy and paste information from your analytic plan here. | _________________________________ | |

| **CDM TABLES AND VARIABLES:**  **Visit the Data Dictionary as a reference while filling out this form. Don't forget to review the "Data Caveats" tab. You will need to log in to Alfresco™ to access.** |
| --- |

| **SCREENINGS** |  |
| --- | --- |
| Specific Types of Scans to Include:  Ex: All Baseline screens, CPT Code XXXXX | _________________________________ |
| Please check off the variables you will need from the "Screenings" table for your project:  (If you choose any of the nodule characteristics such as Shape, Density, Size, etc., you will receive this information for ALL Nodules) | |
| (List of variables provided) | |

| **DEMOGRAPHICS** |  |
| --- | --- |
| Please check off the variables you will need from the "Demographics" table for your project: | |
| (List of variables provided) | |

| **CDM TABLES AND VARIABLES:**  **Visit the Data Dictionary as a reference while filling out this form. Don't forget to review the "Data Caveats" tab. You will need to log in to Alfresco™ to access.** |
| --- |

| **ENGAGEMENT** The entire Engagement Table will be provided for all study projects. | |
| --- | --- |
| CENSUS LOCATION/DETAIL  *We will not send geocode* | |
| Please list the "SES variables" you will need for your project:  Example: XXXX | _________________________________ |

| **DEATH** |  |
| --- | --- |
| Please check off the variables you will need from the "Death" table for your project: | |
| (List of variables provided) | |

| **CAUSE OF DEATH** |  |
| --- | --- |
| Please check off the variables you will need from the "Cause of Death" table for your project: | |
| (List of variables provided) | |

| **ENCOUNTERS** | |
| --- | --- |
| Specify what types of encounters you need. If you need all encounters, type: "All Encounters."  Ex:  All ED Visits  All Inpatient Visits All Encounters | _________________________________ |

| Please check off the variables you will need from the "Encounters" table for your project: |
| --- |
| (List of variables provided) |

| **BMI** |  |
| --- | --- |
| If BMI data needs to be collected prior to screening  time period, please specify that time period here.  Ex. 2 years prior to screening time period | _________________________________ |
| Please check off the variables you will need from the "BMI" table for your project: | |
| (List of variables provided) | |

| **CANCER CASE** |  |
| --- | --- |
| Specific Cancers to include:  Example:  Lung Cancer  All Non-Solid Tumors  All Cancers | _________________________________ |
| Please check off the variables you will need from the "Cancer Case" table for your project: | |
| (List of variables provided) | |

| **SMOKING HISTORY** |  |
| --- | --- |
| If smoking data needs to be collected prior to  screening time period, please specify that time period here:  Ex. 2 years prior to screening time period | _________________________________ |
| Please check off the variables you will need from the "Smoking History" table for your project: | |
| (List of variables provided) | |

| **BENEFITS** |  |
| --- | --- |
| If benefits data needs to be collected prior to  screening time period, please specify that time period here: Ex. 2 years prior to screening time period | _________________________________ |
| Please check off the variables you will need from the "Benefits" table for your project: | |
| (List of variables provided) | |

| **CDM TABLES AND VARIABLES:**  **Visit the Data Dictionary as a reference while filling out this form. Don't forget to review the "Data Caveats" tab. You will need to log in to Alfresco™ to access.** |
| --- |

| **PHARMACY DISPENSES** |  | |
| --- | --- | --- |
| Specific Drugs or Drug Classes to include: | _________________________________ | |
| Do you have a list of NDCs that would help in identification of these drugs? |  | Yes |
|  |  | No |
| Please upload NDCs in a text file with the following specifications.  Use the .txt extension for the text file. The first row of the file should contain variable names.  Variable names should start with a letter or underscore and contain only letters, numbers, and underscores. Values should be separated by a pipe (\|), including the variable names in the first row. Values that contain a pipe (\|) should be enclosed in quotes (this will be rare).  (Example provided) | | |
| Please check off the variables you will need from the "Pharmacy Dispenses" table for your project: | | |
| (List of variables provided) | | |
| **PHARMACY ORDERS** |  | |
| Specific Drugs or Drug Classes to include: | _________________________________ | |
| Do you have a list of NDCs that would help in identification of these drugs? |  | Yes |
|  |  | No |
| Please upload NDCs in a text file with the following specifications.  Use the .txt extension for the text file. The first row of the file should contain variable names.  Variable names should start with a letter or underscore and contain only letters, numbers, and underscores. Values should be separated by a pipe (\|), including the variable names in the first row. Values that contain a pipe (\|) should be enclosed in quotes (this will be rare).  (Example provided) | | |
| Please check off the variables you will need from the "Pharmacy Orders" table for your project: | | |
| (List of variables provided) | | |

| **CDM TABLES AND VARIABLES:**  **Visit the Data Dictionary as a reference while filling out this form. Don't forget to review the "Data Caveats" tab. You will need to log in to Alfresco™ to access.** |
| --- |

| **BASELINE CO-MORBID STATUS**  Baseline Co-Morbid Status is captured for 1 year prior to Eligibility Start through Eligibility Start. | |
| --- | --- |
| If you want co-morbid status for any other time period, please enter that here:  ex. 1 year prior to LCS scan |  |
| Please check off the variables you will need from the "Baseline Co-morbid Status" table for your project: | |
| (List of variables provided) | |

| **CDM TABLES AND VARIABLES:**  **Visit the Data Dictionary as a reference while filling out this form. Don't forget to review the "Data Caveats" tab. You will need to log in to Alfresco™ to access.** |
| --- |

| **PROCEDURES**  Visit the Procedure Codes list as a reference while filling out this section. You will need to log in to Alfresco™ to access. | |
| --- | --- |
| Please upload procedure codes in a text file with the following specifications:  Use the .txt extension for the text file. The first row of the file should contain variable names.  Variable names should start with a letter or underscore and contain only letters, numbers, and underscores. Values should be separated by a pipe (\|), including the variable names in the first row. Values that contain a pipe (\|) should be enclosed in quotes (this will be rare).  (Example provided) |  |
| Please check off the variables you will need from the "Procedures" table for your project: | |
| (List of variables provided) | |
| **DIAGNOSES**  Visit the Diagnosis Codes list as a reference while filling out this section. You will need to log in to Alfresco™ to access. | |
| Please upload diagnosis codes in a text file with the following specifications:  Use the .txt extension for the text file. The first row of the file should contain variable names.  Variable names should start with a letter or underscore and contain only letters, numbers, and underscores. Values should be separated by a pipe (\|), including the variable names in the first row. Values that contain a pipe (\|) should be enclosed in quotes (this will be rare). (Example provided) |  |
| Please check off the variables you will need from the "Diagnoses" table for your project: | |
| (List of variables provided) | |

| **PROVIDERS:** | |
| --- | --- |
| List the tables you would like to link to Provider information:  Ex: Screenings Table, Encounters |  |
| Please check off the variables you will need from the "Providers" table for your project: | |
| (List of variables provided) | |

| **FACILITY** | |
| --- | --- |
| Please list the tables you would like to link to Facility information:  Ex. Encounters |  |
| Please check off the variables you will need from the "Facility" table for your project: | |
| (List of variables provided) | |
| Other Data Needed: | |

| **INFUSIONS** |
| --- |
| (List of variables provided) |

| **LDCT ORDERS** |
| --- |
| (List of variables provided) |

| **AFFIRMATION** |
| --- |

|  |  | I affirm that the project lead and the lead statistician/analyst has approved the information  in this form. |
| --- | --- | --- |
| Please enter your initials:  If more than one person is completing the affirmation  please enter all initials separated by commas. | _________________________________ | |

Appendix 5: PROSPR-Lung PARC Proposal Template

This form was programmed into Research Electronic Data Capture (REDCap)^®^. Any individual requesting to utilize PROSPR-Lung data for any reason was required to complete this form and submit in REDCap^®^, thus kicking off the Proposal Access Review Committee (PARC) review process (see Figure 2 in the manuscript).

1. Basic Project Information
2. Name of person submitting this form:
3. Email address of person submitting this form:
4. Project Title:
5. Project Lead First Name:
6. Project Lead Last Name:
7. Project Lead Email:
8. Is Project Lead a PROSPR-Lung investigator?: Yes or No
9. Project Lead Affiliation:
10. Small Writing Group Members' Full Names and Affiliations
11. If applicable, additional Group Members' Full Names and Affiliations
12. Intended Use and Approvals
13. What Project Does this Proposal Fall Under? (Check all that apply. Refer to Project Description form if unsure)

- Project 1
- Project 2
- Project 3
- Project 4
- Other (please specify)

1. Requesting approval to (check all that apply):

- Analyze existing data in the data repository (includes all data we plan to collect)
- Analyze existing data from two or more individual PROSPR-Lung sites
- Develop a conceptual model
- Develop methods
- Not sure/Other (If Other, enter comment): ________

1. Intended use (choose one):

- Manuscript
- Meeting abstract
- Presentation
- Grant application
- Grant funded independently of PROSPR U54
- Other (If Other, enter comment): ________

1. Data to be used from the following sites (check all that apply):

- Site 1
- Site 2
- Site 3
- Site 4
- Site 5
- N/A: Conceptual Paper or No Data Needed

1. Research Question
2. Specific Aims: (Word Count 400 Max):
3. Background/Rationale/Significance – (Word Count 800 Max): *Be sure to include how this proposal relates to the overall mission of PROSPR*.
4. Study type:

- Observational
- Interventional
- Conceptual
- Methodological
- Other (If Other, enter comment): ________

1. Outline of Methods: (Word Count 800 Max):
2. Data and Analysis
3. Brief description of proposed statistical analysis methods- Include power calculations where necessary (Word Count 800 Max):
4. Level of PROSPR-Lung data to be used (Check all that apply):

- Limited aggregate data
- Limited individual level data
- Not applicable
- Other (If Other, enter comment): ________

1. Which variable types do you plan to use (Check all that apply) **You will be asked to specify specific variables for each selected category*

- Demographics
- Census location
- Death
- Cause of death
- Vitals
- Cancer Case
- Pharmacy
- Encounters
- Diagnoses
- Procedures
- Enrollment
- Smoking History
- Screenings
- Providers
- Ordered Procedures
- Referrals
- Benefits
- Labs
- Other

1. Timeline
2. Initiation Date:
3. Planned Completion Date:
4. It usually takes about 2 weeks for proposals to be reviewed. If you need this proposal reviewed sooner, you can request a rush review. Would you like to request a rush review?

- Yes
- No

1. List References (Optional)

Appendix 6: PROSPR-Lung Product Review – Reviewer Response Form

This form was programmed into Research Electronic Data Capture (REDCap)^®^ as part of the Proposal Access Review Committee (PARC) review and approval process. After an individual submitted the PROSPR-Lung Proposal Form (Appendix 5), the PARC Project Manager distributed the Proposal to the 3-5 PARC members, who reviewed the proposal and answered the following questions within REDCap^®^. See Figure 2 in the manuscript for the PARC process.

Initial Review: Proposal Number/Record ID: ____

Today's Date:

Reviewer Name:

Please provide feedback on the following components of the proposal:

Clarity:

Specific Aims:

Background/Rationale/Significance:

General Methods:

Statistical Methods:

Overlap:

Timeline/Feasibility:

Group Membership (Appropriateness of writing group):

Comments not included in other categories:

Summary:

Priority Assessment (Indicate your assessment on this proposal’s priority with regards to impact, etc.):

High

Medium

Low

Recommendation:

Approved with no or minimal concerns

Provisionally approved with request for additional information (but not resubmission of the proposal)

Disapproved with the option to revise and resubmit

Disapproved without the option to revise

If ‘Provisionally Approved’, additional information requested:

If ‘Disapproved with the option to revise and resubmit,’ which of the following sections need to be revised and resubmitted? (Check all that apply):

Specific Aims

Background/Rationale/Significance

General Methods

Statistical Methods

Appendix 7: Site Feedback Survey #1

This survey was programmed into REDCap^®^. Study team members were invited to take this anonymous survey.

**PROSPR-Lung Annual Feedback Survey**

Please let us know how we did last year, and what we can do to be more effective. This anonymous survey should take 15 minutes or less to complete. We appreciate your time and feedback!

What is your role in PROSPR-Lung? (choose all that apply)

□ Investigator

□ PM

□ Data Manager/Coordinator

□ Programmer

□ Abstractor

□ Research Assistant

□ NCI or PCC

□ Other: __________________

□ Prefer not to answer

How long have you been a part of PROSPR-Lung?

□ Since inception (late 2016)

□ 7-12 months

□ 1-6 months

□ <1 month

**Contracts**

I wish to leave feedback about the Contracting process

□ Yes

□ No

How did the contracting process go for you?

□ Great

□ Better than fine, less than great

□ Fine

□ Better than terrible, but not fine

□ Terrible

What worked well? What could be improved?

**Communications**

I wish to leave feedback about the lead site’s communications.

□ Yes

□ No

How do you feel about the frequency of communications?

□ Too frequent

□ Just right

□ Too infrequent

How do you feel about the content of communications? [Open text field]

How do you feel about the clarity of communications? [Open text field]

What additional feedback or suggestions for improvement do you have regarding communications from the lead site?

**Meetings**

I wish to leave feedback about PROSPR-Lung meetings.

□ Yes

□ No

Please indicate which meetings that you regularly attend. For each meeting selected, please answer the following:

□ How do you feel about the frequency this meeting?

□ Too frequent

□ Too infrequent

□ Just right

□ Other (explain):

How do you feel about the content of this meeting?

□ Lacking

□ Overly packed

□ Just right

□ Other (explain):

How do you feel about the pace of this meeting?

□ Too slow

□ Too fast

□ Just right

□ Other (explain):

What additional feedback or suggestions for improvement do you have regarding meetings?

**Data**

I wish to leave feedback about the Data Acquisition Unit

□ Yes

□ No

Do you find the work plans to be easy to follow?

□ Yes

□ No

If No, please explain and provide suggestions for improvement

Do you feel that you understand the Abstraction plans?

□ Yes

□ Somewhat

□ No

If Somewhat or No, please explain and provide suggestions for improvement:

What additional feedback or suggestions for improvement do you have regarding PROSPR-Lung data management?

**Additional Comments**

Please use this space to give us any additional thoughts/feedback:

OPTIONAL: If you have left specific feedback that you would like a direct response to, please enter your email address here and a study team member will be in touch with you:

Please also indicate what specific sections you would like a direct response to.

Appendix 8: Site Feedback Survey #2

This survey was programmed into REDCap^®^. Study team members were invited to take this anonymous survey.

**PROSPR-Lung Annual Feedback Survey**

Please let us know how we did last year, and what we can do to be more effective. This anonymous survey should take 15 minutes or less to complete. We appreciate your time and feedback!

What is your role in PROSPR-Lung? (choose all that apply)

□ Investigator

□ PM

□ Data Manager/Coordinator

□ Programmer

□ Abstractor

□ Research Assistant

□ NCI or PCC

□ Other: ________________________

□ Prefer not to answer

**Data Acquisition Activities**

We are asking for your feedback on data acquisition activities since we last surveyed you in November 2018. Would you like to leave feedback on these?

□ Yes

□ No

Do you feel that the topics covered in the programmer meetings are valuable?

□ Yes, strongly agree

□ Yes, agree

□ Neutral

□ No, disagree

□ No, strongly disagree

Suggestions for improvement:

How do you feel about your involvement in creating agendas for programmer meetings?

□ Too much

□ Just right

□ Too little

□ Other (Please explain):

Suggestions for improvement:

How do you feel about the frequency of the programmer meetings?

□ Too frequent

□ Just right

□ Too infrequent

□ Other (Please explain):

How clear do you find the workplans?

□ Extremely clear

□ Somewhat clear
□ Neutral

□ Somewhat unclear
□ Extremely unclear

Suggestions for improvement:

Do you feel like you have sufficient opportunity to provide feedback on the workplans?

□ Too much

□ Just right

□ Too little

□ Other (Please explain):

Suggestions for improvement:

How clear are deadlines/due dates for data activities?

□ Extremely clear

□ Somewhat clear
□ Neutral

□ Somewhat unclear
□ Extremely unclear

Suggestions for improvement:

How do you feel about your involvement in providing feedback on the timeline for data/programming activities?

□ Too much

□ Just right

□ Too little

□ Other (Please explain):

Suggestions for improvement:

How do you feel about your involvement in data QA?

□ Too much

□ Just right

□ Too little

□ Other (Please explain):

Suggestions for improvement:

Do you feel that you understand why data tasks are being assigned to your site?

□ Yes, strongly agree

□ Yes, agree

□ Neutral

□ No, disagree

□ No, strongly disagree

Suggestions for improvement:

In general, do you understand what the DAU is asking your site to do?

□ Yes, strongly agree

□ Yes, agree

□ Neutral

□ No, disagree

□ No, strongly disagree

Suggestions for improvement:

In general, do you find that you understand how data tasks are related to one another?

□ Yes, strongly agree

□ Yes, agree

□ Neutral

□ No, disagree

□ No, strongly disagree

Suggestions for improvement:

How do you feel about the extent to which you are cc'd on data-related communications to your site from the DAU?

□ I am cc'd too often

□ I am cc'd the right amount

□ I am not cc'd enough

Please comment on anything else not covered in the above questions.

**PROSPR-Lung Communications**

I wish to leave feedback about the lead site’s communications. This includes all email communications coming from the lead site (data and non-data related).

□ Yes

□ No

How do you feel about the frequency of communications?

□ Too frequent

□ Just right

□ Too infrequent

Suggestions for improvement:

**PROSPR-Lung Steering Committee and/or In-Person Meetings**

I wish to leave feedback about PROSPR-Lung Steering Committee and/or In-person meetings.

□ Yes

□ No

How do you feel about the frequency of the PROSPR-Lung Steering Committee meetings?

□ Too frequent

□ Just right

□ Too infrequent

□ Other (Please explain):

Do you feel that the topics covered in the PROSPR-Lung Steering Committee meetings are valuable?

□ Yes, strongly agree

□ Yes, agree

□ Neutral

□ No, disagree

□ No, strongly disagree

Suggestions for improvement:

Do you feel that we cover an appropriate number of topics in the PROSPR-Lung Steering Committee meetings?

□ No, we try to cover too much

□ Yes, just right

□ No, we don't cover enough

Suggestions for improvement:

Overall, do you find the PROSPR-Lung Steering Committee meetings engaging?

□ Yes

□ No

If no, what would make the PROSPR-Lung Steering Committee meetings more engaging for you?:

How do you feel about the frequency of the PROSPR-Lung in-person meetings?

□ Too frequent

□ Just right

□ Too infrequent

□ Other (Please explain):

Do you feel that the topics covered in the PROSPR-Lung in-person meetings are valuable?

□ Yes, strongly agree

□ Yes, agree

□ Neutral

□ No, disagree

□ No, strongly disagree

Suggestions for improvement:

Do you feel that we cover an appropriate number of topics in the PROSPR-Lung in-person meetings?

□ No, we try to cover too much

□ Yes, just right

□ No, we don't cover enough

Suggestions for improvement:

Overall, do you find the PROSPR-Lung in-person meetings engaging?

□ Yes

□ No

If no, what would make PROSPR-Lung in-person meetings more engaging for you?:

What additional feedback and/or suggestions for improvement do you have regarding PROSPR-Lung meetings?

**Additional Comments**

Please use this space to give us any additional thoughts/feedback:

OPTIONAL: If you have left specific feedback that you would like a direct response to, please enter your email address here and a study team member will be in touch with you:

Please also indicate what specific sections you would like a direct response to.
